# Cardiac-vagal rhythm echoes on the heartbeat’s mechanosensory imprint in the brain

**DOI:** 10.1038/s42003-025-08969-x

**Published:** 2025-11-17

**Authors:** Diego Candia-Rivera, Mario Chavez

**Affiliations:** Sorbonne Université, Institut du Cerveau—Paris Brain Institute—ICM, CNRS, Inria, Inserm, AP-HP, Hôpital de la Pitié Salpêtrière, Paris, France

**Keywords:** Computational biophysics, Neuro-vascular interactions, Cardiovascular biology

## Abstract

The heart, a highly reactive and innervated organ, plays a crucial role in brain-viscera communications. Recent research has highlighted the role of mechanosensation in the brain, where ion channels in neurons’ membranes respond to heartbeat-induced pressure changes, triggering specific neural responses. Cardiac mechano-electric coupling ensures cardiac output to match venous return through beat-by-beat feedback. However, the effect of ongoing cardiac rhythms on the brain-sensed strength of each heartbeat is not well understood. This is crucial for exploring brain-heart communication pathways and for understanding the mutual influence between brain and cardiac oscillations. This study explores how cardiac rhythms influence heartbeat strength (HBS) as detected by the brain in humans. As a proxy for brain-sensed HBS, we used ballistocardiographs, which capture HBS from the back of the head while participants are in horizontal position. By modeling HBS, we demonstrate that fast fluctuations in heart rate variability significantly influences the final HBS. This suggests a direct relationship between vagal tone and subsequent neural responses to heartbeats, highlighting the necessity of studying visceral oscillations in the context of mechanosensation and inter-organ communication research.

## Abstract (web summary)

Vagal-driven heart rhythm fluctuations modulate the heartbeat strength sensed in the head, linking cardiac rhythms to brain mechanosensation. These findings highlight vagal tone’s role in shaping brain-heart communication.

## Introduction

The heart is highly reactive to the body's milieu. It is considered one of the main channels of communication between the brain and the viscera^1^. Evidence of brain-heart communication has primarily emerged by reports of brain responses to heartbeats^2^, brain correlates of cardiac autonomic outflow^3^ and the modeling of mutual influences between brain and cardiac oscillations^1^. Specifically, brain responses to heartbeats have been reported in different sensory hubs across the brain: the somatosensory cortex, ventromedial prefrontal cortex, posterior cingulate cortex/ventral precuneus, insula, frontal operculum, parietal lobule and extrastriate visual cortices (see Engelen et al.^2^ for a review). It is important to note that the brain-heart communication also occurs through non-neural pathways, including immunologic, metabolic, and hormonal mechanisms^4^. Recent research has provided insights into brain-heart communication via mechanosensation mechanisms, in which membrane ion channels, such as PIEZO2, sense changes in neuron membrane pressure caused by heartbeat pulsations, triggering neural responses in the brain^5^. This newly described pathway offers promising research avenues to understand what is the function of the mechanosensitive pathway^6^ and the specific role of cardiovascular biophysics^7^. While research focused on brain responses to heartbeats (also called heartbeat-evoked responses or heartbeat-evoked potentials) systematically attempts to prove that the observed effects are not driven by cardiovascular effects such as heart rate, recent findings point to direct influences from stroke volume and blood pressure^8–10^. Those effects have been hypothesized to be part of the ongoing feedback loops between central and autonomic nervous systems^9^. Additionally, evidence supports the existence of low-frequency cardiovascular rhythms of central origin, which may contribute to these loops. Such rhythms, originating from brainstem pacemaker-like activity^11,12^, and their neurovascular interactions^13^, suggest that oscillatory dynamics may play a key role in mediating brain-body communication at multiple timescales.

Heartbeat pulsation occurs throughout the entire body, varying across regions and over time^14^. Abnormal pulsation is typically assessed in the context of cardiovascular diseases, hydrocephalus, or traumatic brain injury^15,16^. However, fluctuations in pulsation strength also occur in healthy individuals, much like other cardiovascular variables such as heart rate^3^. While such variability is recognized as part of the body’s ongoing autonomic regulation by the sympathetic and parasympathetic nervous systems^17^, whether there is a relationship between heartbeat pulsation strength in the brain and cardiac rhythmicity remains to be investigated. In the context of brain-heart communication, clarifying this relationship is crucial to better understand the interplay between brain-heart parallel rhythms, neural responses to heartbeats, and potential brain responses that depend on pulsation strength.

Cardiac rhythmicity is typically quantified from both slow and fast fluctuations in heart rate variability (HRV), which are indirect markers of adrenergic and cholinergic neurotransmitter effects^18,19^. These neurotransmitters cause electrical excitation in the sinoatrial node, leading to the heartbeat^20,21^. Cardiac control occurs through the mechano-electric coupling, involving beat-by-beat feedback from the local mechanical environment to electrical activity, forming an autoregulatory loop^22^. So far, the main attribution to this mechano-sensitivity is to ensure that cardiac output matches venous return, maintaining balanced cardiovascular performance.

While mechano-electric coupling explains its relationship with changes in heart rate^23,24^, little is known about how ongoing heart rhythms influence heartbeat strength (HBS). Modeling often overlooks factors like cardiac rhythmicity that influence HBS. When these factors are considered, they are typically limited to experimental conditions involving physical effort^25^, where cardiac rhythmicity is highly reduced^26^.

**Fig. 1:**
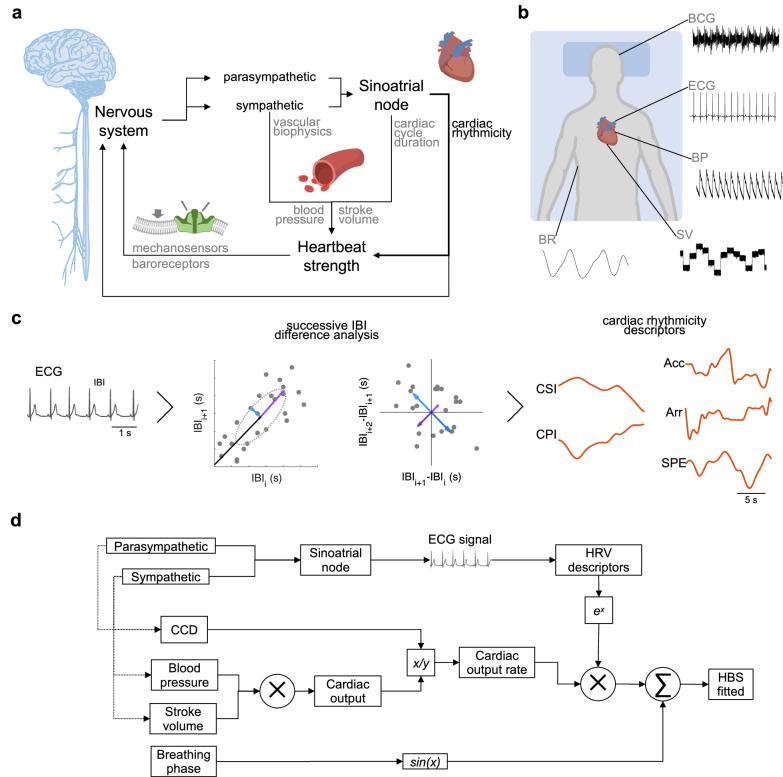
Factors influencing the sensed heartbeat strength. **a** Theoretical schematic of the factors involved, encompassing sympathetic influences in vascular properties, and sympathetic and parasympathetic influences in cardiac rhythmicity, that together influence the heartbeat strength (HBS), sensed at central level by baroreceptor neurons and mechanosensitive ion channels in neuron membranes. **b** Experimental setup. Participants remained in horizontal position for approximately 400 s, while being recorded ballistocardiogram (BCG), electrocardiogram (ECG), stroke volume (SV), blood pressure (BP) and breathing patterns (BR). **c** Computation of cardiac descriptors from inter beat interval (IBI) analyses, using first order (IBI_i_ vs. IBI_i+1_) and second order (IBI_i+1_ − IBI_i_ vs. IBI_i+2_ − IBI_i+1_) Poincaré plots, to gather cardiac parasympathetic index (CPI), cardiac sympathetic index (CSI), heart rate acceleration-deceleration balance (Arr), heart rate arrhythmic behavior (Arr), and second order Poincaré plot entropy (SPE). **d** Mathematical model of the HBS. Parasympathetic and sympathetic influences heartbeat generation. First and second-order cardiac descriptors are generated from the generated ECG. In parallel, sympathetic activity influences blood pressure and stroke volume. These variables are combined to fit a function of HBS, that is adjusted with a least squares optimization, based on the empirical HBS.

The sympathetic nervous system plays a crucial role in regulating cardiovascular function by influencing the vascular properties and, consequently, the strength of the heartbeat. Sympathetic activations trigger the release of adrenergic neurotransmitters^18^, which bind to receptors on vascular smooth muscle cells, causing them to contract and increase vascular resistance. This constriction of blood vessels raises blood pressure^27^, which requires the heart to pump more forcefully to maintain adequate blood flow. As a result, the strength of the heartbeat is enhanced to overcome the increased resistance and ensure efficient circulation throughout the body (Fig. 1a). Cardiac contractility, an indirect measure of HBS, refers to how well the heart can pump blood with each beat, considering the pressure in the arteries and the volume of blood in the heart before it contracts^28^. Measures of contractility include the fraction of blood pumped out with each beat (stroke volume) and the speed and pressure of blood flow.

The impact of parasympathetic activity and its interaction with sympathetic activity on HBS is still not well understood. Some studies have explored the relationship between the stretch of the sinoatrial node and vagal tone^29^ (which is related to high-frequency HRV), but this area remains largely unexplored. This understanding is now crucial in the context of brain-heart communication, where mechanosensation has been shown as a pathway in brain-viscera communication^5^, and the abundant evidence underscoring the role of parasympathetic/vagal activity in brain-viscera dynamics^30^.

Given the existing gap between heartbeat mechanosensation in the brain and ongoing changes in cardiac rhythmicity, and considering the apparent feedback loops between neural responses to heartbeats and cardiovascular parameters^9^, this study aims to show that cardiac rhythms influence the strength of heartbeats as perceived by the brain. To estimate brain-sensed HBS, we used ballistocardiography, recording pulsations from the back of the head while participants were in a horizontal position. By modeling HBS, we found that fast fluctuations in heart rate variability significantly contribute to explaining the changes over time in pulse mechanosensation. Meaning that instantaneous heart rhythms could influence the pulsation strength in the head. These findings suggest a potential link between ongoing vagal tone and neural responses to heartbeats.

## Results

### Modeling of cardiovascular biophysics

In this study, we explored whether spontaneous fluctuations in cardiac rhythms can contribute to better explain ongoing fluctuations in HBS sensed at the brain level.

To study the relationships between cardiovascular dynamics and the consequent fluctuations in the brain-sensed HBS, we studied noninvasive measures of HBS at the head level, through ballistocardiographs^14^. Concurrent measures included electrocardiogram, breathing patterns, stroke volume and blood pressure (Fig. 1b).

Using the electrocardiogram, we gathered measures of HRV, namely cardiac rhythmicity descriptors, which describe different patterns of activations of sympathetic or parasympathetic activities. The sympathetic nervous system affects heart rate and rhythm through adrenergic mechanisms, while the parasympathetic nervous system does so through cholinergic mechanisms. Additionally, cardiac descriptors can capture how these systems shift over time, showing how they switch roles or dominate at different moments. Therefore, HRV was characterized here by five parameters, as depicted in Fig. 1c. First, by capturing differences in inter-beat intervals (IBI) using the Poincaré plot. This allows us to derive measures of rhythmicity by quantifying successive differences in IBI (IBI_i_ vs. IBI_i+1_, *i* is an integer)^31^: the cardiac sympathetic index (*CSI*) capturing slow fluctuations in HRV, the cardiac parasympathetic index (*CPI*) capturing fast fluctuations in HRV. Second, by deriving measures of rhythmicity by quantifying changes in groups if three consecutive IBI (IBI_i+1_ − IBI_i_ vs. IBI_i+2_ − IBI_i+1_, *i* is an integer)^32^: the heart rate acceleration-deceleration balance (*Acc*) capturing the consecutive increases in heart rate with respect to the decreases in a defined time scale, the heart rate arrhythmic behavior (*Arr*) capturing consecutive increase-decrease and decrease-increase in heart rate, and the second-order Poincaré entropy (*SPE*) capturing the complexity of dynamic shifts of increase-decrease in heart rate.

As shown in Fig. 1d, we aim at drawing relationships between different measures of sympathetic and parasympathetic nervous systems, and how they ultimately relate to the head-sensed HBS. On one hand, fluctuations in sympathetic and parasympathetic influence heartbeat generation^20^, on the other hand, the sympathetic influences on vascular properties. Our model considers the stroke volume and blood pressure as direct determinants of the force of each heartbeat. The product of stroke volume and blood pressure reflects the cardiac output per beat, which is a key determinant of the force of each heartbeat, while cardiac cycle duration (CCD) defines the rate of the cardiac output (a longer duration means less frequent beats), impacting the relative strength of each beat. We employ time-resolved descriptors of cardiac rhythmicity^31^ and explore their role in the ongoing changes in HBS.

The model considers blood pressure (BP) and stroke volume (SV) directly influencing HBS. We assumed that HRV causes a potential damping effect on HBS^33^, which was modeled as an exponential function. In addition, we included potential influences of the breathing cycle (breathing phase, ${\phi }_{{BR}}$), to consider potential remnants of breathing pattern artifacts in the measured HBS. Therefore, we modeled HBS as:

1 $${{HBS}}\left(t\right)={c}_{1}\cdot \left(\frac{{SV}\left(t\right)\cdot {{BP}}\left(t\right)}{{{CCD}}\left(t\right)}\right)\cdot {e}^{\,{-c}_{2}{{HRV}}\left(t\right)}+{c}_{3}\cdot \sin \left({\phi }_{{{BR}}}\left(t\right)\right)$$

where ${c}_{1}$, ${c}_{2}$, and ${c}_{3}$ are constants adjusting individual factors’ influence in the model: ${c}_{1}$ adjust the overall amplitude, ${c}_{2}$ the damping effect of HRV and ${c}_{3}$ the amplitude of the remnant breathing pattern artifacts. The coefficients ${c}_{1}$, ${c}_{2}$, and ${c}_{3}$ from Eq. (1) were fit and adjusted per each participant, using a non-linear least squares optimization.

### Cardiac vagal rhythm exhibits the strongest association with heartbeat strength

We validated our model exploring the relationship between cardiac rhythms and their influence on the motion caused by pulsations at the head level, as measured from ballistocardiography^14^.

**Fig. 2:**
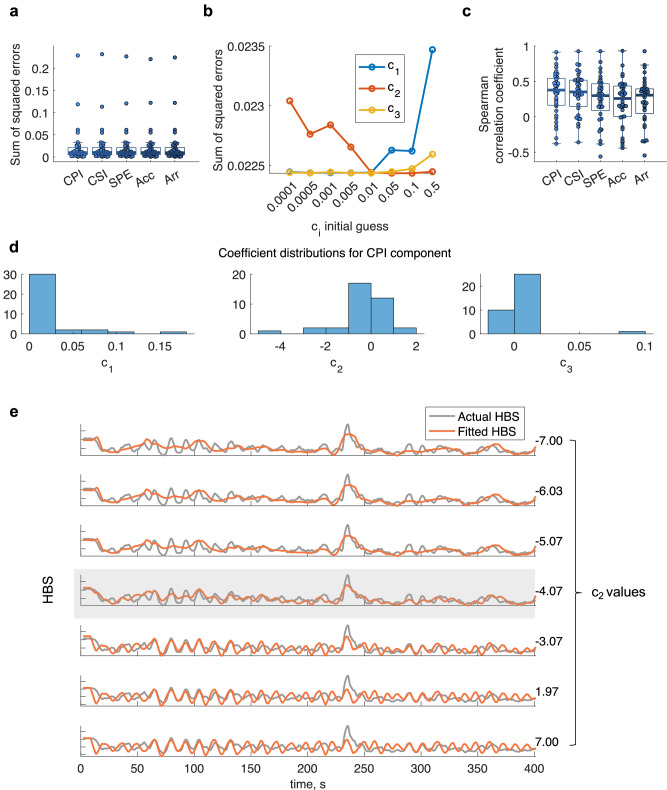
Results on the heartbeat strength (HBS) model fitting. **a** Sum of squared errors between the measured HBS and the fitted HBS, when using as heart rate variability (HRV) term: cardiac parasympathetic index (CPI), cardiac sympathetic index (CSI), second-order Poincaré plot entropy (SPE), heart rate acceleration-deacceleration balance (Acc), and heart rate arrhythmic behavior (Arr). Box plots indicate the group median and 25–75 percentiles. **b** Group mean sum of squared errors of the HBS fitting, using as HRV input the CPI, as a function of the initial guess of c_1_, c_2_ and c_3_. **c** Spearman correlation coefficients between the measured HBS and the fitted HBS. Box plots indicate the group median and 25–75 percentiles. **d** Distribution of the coefficients fitting the HBS model, incorporating CPI as the HRV term. **e** Single participant example on the effect of changing c_2_, the coefficient of the HRV damping effect in the HBS fitting. The gray shaded are indicates the identified optimal c_2_ using the least squares optimization.

The model of HBS was applied to various time series that track sympathetic and parasympathetic activations using time-varying measures of HRV. As shown in Fig. 2a, b, CPI performed the best in fitting the model, based in overall fitting metrics. On average, CPI showed the lowest sum of square errors (SSE; SSE_CPI_ = 0.0224, SSE_CSI_ = 0.0226, SSE_SPE_ = 0.0227, SSE_Acc_ = 0.0226, SSE_Arr_ = 0.0227). Figure 2b shows that the initial guess of the coefficients c_1_, c_2_ and c_3_ does not significantly modulate the SSE fitting values. As an additional indicator of goodness-of-fit, we computed Spearman correlation coefficients between the modeled HBS and the measured HBS (Fig. 2c). On average, CPI showed the highest Spearman correlation coefficient (ρ; ρ_CPI_ = 0.3349, ρ_CSI_ = 0.3313, ρ_SPE_ = 0.2452, ρ_Acc_ = 0.2059, ρ_Arr_ = 0.2328). Note that although most participants showed a good fit in modeling HBS, some exhibited negative correlation coefficients. This is mainly due to accurate amplitude modeling paired with poor phase alignment. Although we examined complex patterns of cardiac rhythmicity (Acc, Arr, SPE), our findings indicate that HBS is primarily driven by cardiac rhythms themselves, rather than by complex shifts between sympathetic and parasympathetic activity, which were measured in this study as distinct transition patterns between heart rate accelerations and decelerations.

These results suggest that cardiac vagal tone may play a crucial role in understanding HBS. Figure 2d displays the distribution of the model coefficients across participants. Interestingly, c_2_ presents both positive and negative values: c_2_ > 0 exhibits damping, meaning that the amplitude of oscillations or the response decreases over time, as it causes the exponential function to decay as time increases, leading to a reduction in the magnitude of the response, indicative of energy dissipation. Instead, c_2_ < 0 exhibits growth or instability, meaning that the amplitude of oscillations increases over time. The resulting positive exponent causes the exponential function to grow as time increases, leading to an increase in the magnitude of the response, rather than indicating an unstable system where HRV is adding energy. This can reflect the presence of potential feedback loops, causing the oscillations to increase over time.

Figure 2e illustrates the influence of the cardiac vagal tone (CPI) in a single subject, demonstrating how changes in the contribution of this component are related to variations in the modeling of the HBS at the head level.

### Heartbeat strength modeling is significantly enhanced when incorporating heart rhythms

**Fig. 3:**
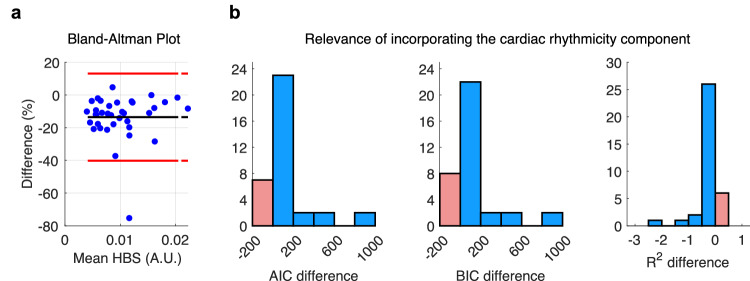
Heartbeat strength (HBS) model fitting evaluation, using CPI as the HRV term. **a** Bland-Altman plot showing the distribution of the percentual error in the estimation of HBS, as a function of the mean measured HBS. **b** Evaluation of excluding the HRV term in the HBS model fitting. *R*^2^ difference < 0 indicates that HRV improves the model, and AIC or BIC difference > 0 indicates that HRV improves the model. Blue bars indicate the cases where there was an improvement by incorporating HRV, and red bars indicate the cases where there was not an improvement.

To evaluate the sensitivity of the model, we examined whether specific patterns in HBS led to types of errors in the model’s fitting. Figure 3a displays the Bland-Altman plot for the HBS model fitted using CPI, which shows the percentage error of the model in relation to the mean of the empirical HBS. Our analysis reveals that the model’s error does not increase systematically with any pattern of HBS, as measured by these statistical metrics.

To verify whether including the HRV component improves the model, we compared it with a simplified model that excludes HRV, as shown in Eq. (2):

2 $${{HBS}}\left(t\right)={c}_{1}\cdot \left(\frac{{{SV}}\left(t\right)\cdot {{BP}}\left(t\right)}{{{CCD}}\left(t\right)}\right)+{c}_{2}\cdot \sin \left({\phi }_{{{BR}}}\left(t\right)\right)$$

First, we observed that the *R*^2^ value was lower for the model without HRV, indicating that including HRV enhances the model’s accuracy (Fig. 3b). To ensure that this improvement is not simply due to the HBS model based on a more complex HRV description (with more parameters), we also used the AIC and BIC criteria. These criteria adjust for the number of parameters to provide a fair comparison. We found that both AIC and BIC values significantly increased when HRV was excluded, suggesting that omitting HRV worsens the model fit in most subjects. Overall, these results confirm that including the HRV component significantly improves the estimation of HBS, demonstrating a direct relationship between vagal tone and HBS.

**Fig. 4:**
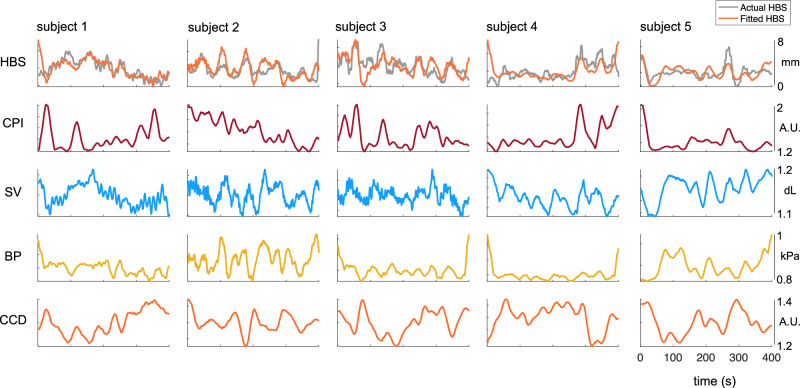
Parallel fluctuations of heartbeat strength and cardiovascular parameters. Five examples of the heartbeat strength (HBS) modeling using cardiac parasympathetic index (CPI), stroke volume (SV), blood pressure (BP), and cardiac cycle duration (CCD).

Figure 4 presents five examples illustrating both the empirical and modeled HBS, alongside measurements of vagal tone (CPI), stroke volume (SV), blood pressure (BP), and cardiac cycle duration (CCD). These examples illustrate that the modeled HBS is influenced by the interaction of all these cardiovascular variables, rather than any single one. These results demonstrate that cardiovascular biophysics, heart rate, and rhythm collectively play a key role in capturing the dynamic fluctuations of heartbeat mechanosensation. Incorporating all these elements together is essential for accurate modeling.

### Cardiac-vagal rhythm does not reflect an effect of spontaneous changes in breathing patterns

**Fig. 5:**
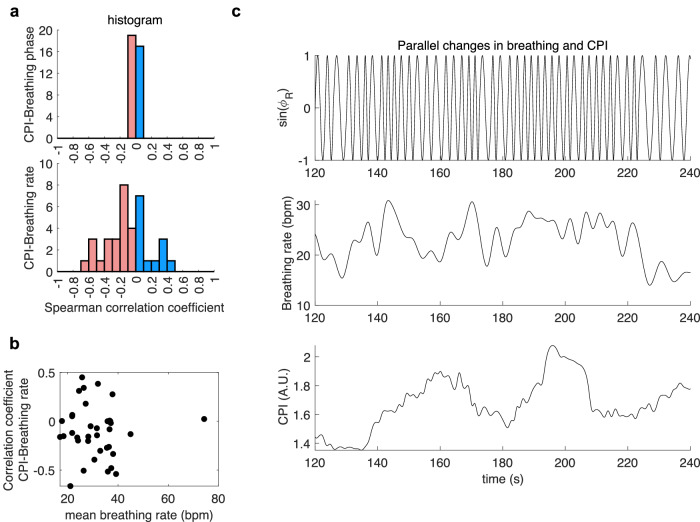
Relationships between the cardiac parasympathetic index (CPI) and breathing phase (ϕ_R_) and rate (in breaths per minute, bpm). **a** Histogram of the Spearman correlation coefficients between CPI and breathing pattern (sin(${{{\rm{\phi }}}}_{{{\rm{R}}}}$)) and breathing rate. Red indicates a negative correlation coefficient, and blue indicates the positive ones. **b** Distribution of the Spearman correlation coefficient between CPI and breathing rate with respect to the mean breathing rate. Each data point is one participant. **c** Example of the parallel changes in breathing pattern, rate and CPI for one participant.

To assess the specificity of the CPI in capturing physiological dynamics beyond those solely driven by respiration, we conducted an analysis quantifying the degree of respiratory sinus arrhythmia during the task. Specifically, Spearman correlation coefficients were computed between CPI and respiratory activity for each participant. Figure 5a presents the distribution of these coefficients with respect to both breathing pattern and breathing rate. Overall, no consistent or systematic correlation was observed. While some participants exhibited absolute correlation coefficients as high as 0.5, neither the magnitude nor the direction of these associations appeared to be related to the individual’s average breathing rate during the task (Fig. 5b). For illustrative purposes, Fig. 5c displays the temporal profiles of CPI and respiratory activity for a representative participant. The figure highlights that the timescale of breathing pattern fluctuations differs markedly from that of CPI, supporting the conclusion that CPI does not directly reflect spontaneous variations in breathing rate.

## Discussion

In this study, we investigated the relationship between cardiac rhythms and HBS as detected in the head in humans. We found that cardiac vagal rhythm (quantified as CPI) is significantly related to spontaneous fluctuations in heartbeat pulsation strength in the head.

The parasympathetic nervous system, in contrast to the sympathetic nervous system, primarily works to promote relaxation and reduce cardiovascular activity. It achieves this by releasing acetylcholine, which acts on receptors in the heart to slow down the heart rate, and potentially decreasing the force of each heartbeat. This results in reduced cardiac output and blood pressure. By causing blood vessels to dilate and lowering the heart’s workload, the parasympathetic nervous system helps maintain a balanced state of rest and recovery, counteracting the stimulating effects of the sympathetic nervous system.

Our results indicate that, beyond these linear relationships, the vagal tone actively influences the sensed HBS in the head, by either causing dampening effects or by introducing energy. Our results provide new insights into the role of the parasympathetic nervous system in the fluctuations in HBS, which knowledge was limited to indirect links with the stretch of the sinoatrial node^29^ or the amplitude of the R-peak in the ECG^34^.

The vagus nerve plays a key role in the bidirectional communication between the brain and other organs, mediating and influencing a wide range of physiological functions and behavior. As the main component of the parasympathetic nervous system, the vagus nerve functions encompass influences in heart rate, blood pressure, breathing, and gut motility (Fig. 1a), while also coordinating complex reflexes and behaviors like swallowing, coughing, and feeding^30^.

The emerging evidence suggests that vagal pathways are not merely passive conduits for sensory information but actively engage in complex computations that shape the body’s internal state and its interactions with the environment^35^. Vagal sensory neuron subtypes innervate various organs^36–39^, mediating interorgan communication, which underscores the complexity of this system. These neurons exhibit distinct response properties and engage different neural circuits depending on the organ they innervate—be it the heart, lungs, or gut^30^. The complex signal transduction mechanisms employed by these neurons remain poorly understood. However, the variety in sensory neuron types suggests that the vagus nerve integrates a multitude of signals to maintain homeostasis and respond dynamically to environmental and internal changes.

Recent hypotheses propose that the vagus nerve engages in active signal processing, similar to other sensory systems like the visual or olfactory systems^35^. This perspective suggests that vagal sensory neurons compute spatial and temporal features of viscerosensory inputs, rather than merely relaying information to the brain. This would allow the interplay of ascending sensory and descending motor signals, challenging the traditional view of strict segregation between sensory and motor pathways. Such a mechanism could enable the vagus nerve to finely tune physiological responses, enhancing metabolic efficiency and promoting allostasis—an active process of anticipating and adapting to changes to maintain internal stability^40^.

The brain monitors internal bodily signals through complex interoceptive networks, influencing various neural processes, including multisensory integration and cognition^2^. While the exact functional role of the HBS and consequent mechanical sensing at the brain level is not fully understood, evidence suggests this pathway enables a quicker relay of bodily signals to specific brain regions, as compared to synaptic firings^41^. These cardiovascular dynamics may allow synchronizing heartbeat dynamics with a specific brain network, allowing synchronization between distant nodes in complex physiological networks^1^.

The vagus nerve is involved in the regulation of inflammation, and the potential of vagus nerve stimulation for therapeutic interventions encompasses epilepsy, rheumatoid arthritis, depression, and headaches^42^. However, vagus nerve stimulation is highly unspecific, presenting challenges in precisely targeting therapeutic effects without inducing unwanted side effects. Understanding the specific computational roles of different vagal fibers could lead to more refined approaches, potentially enabling selective modulation of desired pathways for therapeutic benefit.

Beyond brain-heart communication, mechanosensation has an important role in cardiovascular regulation^43^. For instance, Piezo1 channels are essential for blood vessel formation, valve development, and regulating blood vessel tone and baroreceptor reflex. When activated by high pressure, Piezo1 can disrupt lung blood vessel barriers and remodel arteries in hypertension. Understanding mechanosensation functions can help develop new treatments for vascular diseases like atherosclerosis and hypertension.

Understanding how cardiac changes influence ongoing heart mechanical properties can provide insights into cardiovascular health and disease; for instance, it might help in predicting the risk of cardiac events or tailoring interventions in patients with heart conditions. For instance, in heart failure a reduced contractility leads to lower stroke volume and can result in low blood pressure, or in hypertension, where chronic high blood pressure can be due to increased peripheral resistance and sometimes increased contractility^44^, necessitating interventions to reduce the workload on the heart.

The main limitation of this study is that it focuses on modeling large-scale features, overlooking the rich cellular mechanisms that drive cardiac contractility. At the cellular level, the main factor influencing contractility is how well the molecular motors (myosin cross-bridges) in the sarcomeres generate tension and shorten. This is affected by the rates and extent of calcium activation, the turnover of the cross-bridges, and how responsive the sarcomeres are to calcium^45^. However, the inclusion of cardiac rhythmicity may partially cover some of these hidden dynamics by signaling the regulatory pathways that control contractility as they are activated by the autonomic nervous system, encompassing the potential influences of other factors, such as pH or temperature.

Another limitation of this study is that measurements of blood pressure and stroke volume relayed on model-based measures instead of empirical measurements, which could reduce the accuracy of our HBS modeling.

Finally, our results revealed an association between parasympathetic (vagal) activity and the sensing of HBS. While parasympathetic dynamics are often, but not exclusively, linked to breathing patterns, our analyses showed no significant correlation between the two signals. Although we cannot entirely exclude a contribution from breathing, the evidence presented in this study suggests that the observed effects are largely independent of respiratory influences.

The mechanosensitive communication between the brain and heart represents a compelling and necessary avenue of research^6^, crucial not only for understanding its physiological roles but also its implications in cognition^46^. A growing body of literature has shown that neural responses to heartbeats contribute to perceptual awareness and are disrupted across a range of neurological and psychiatric conditions^47^. While the heart is known to convey information through the shape, timing, and rhythm of its cycle, it can also be hypothesized that the strength of each heartbeat may carry additional functional significance. However, the mechanisms underlying fluctuations in HBS remain poorly understood, including its differential sensing across body regions. Our findings emphasize the importance of considering cardiac rhythms in this context, as they account for some variance in HBS that is not explained by blood pressure, stroke volume, or breathing. Future research could investigate whether targeting autonomic neuromodulation might offer therapeutic avenues for pathological intracranial pulsatility in clinical settings^16^. From a broader physiological perspective, this research can contribute to further comprehend mechanosensation in other systems, including baroreception^48^, gastrointestinal motility^49^, and cutaneous perception^50^. In addition, further research is needed to better understand the oscillatory patterns of feedback loops between autonomic and central systems, which may support information integration between physiological and neural domains. Specifically, whether cardiovascular oscillations precede neural ones^13,51^, suggesting temporally structured coordination that future mechanistic models of brain-heart interaction should incorporate.

## Conclusion

In conclusion, the interaction between the sympathetic and parasympathetic branches of the autonomic nervous system can affect both heart rate variability and the strength of the heartbeat. Our work is one of the first attempts in further elucidating mechanical properties of the heartbeat, in resting awake individuals, further elucidating the biophysics of brain-heart coupling.

Importantly, we found that cardiac parasympathetic activity has a leading role in the fluctuations of the brain-sensed HBS. This sums to the critical role of the vagus nerve as mediator of brain-viscera communication, with its sensory neurons playing an active role in computing and integrating signals that regulate autonomic functions and behavior. Future research aimed at elucidating the mechanisms underlying vagal computations, mechanisms and their role in disease could open new avenues for targeted therapies, enhancing our ability to treat a range of disorders that involve dysregulation of the autonomic nervous system.

## Materials and methods

### Study cohort

Data were collected from 40 adult participants (17 male), without specific inclusion criteria. For the purposes of this study, 4 from them were not included for presenting history of cardiovascular conditions. Participant ages ranged 18–65 years, and their body mass indices (BMIs) ranged 18–48 kg/m^2^. The competent ethics committee approved the study (Kansas State University, IRB 9386). All participants gave their consent to participate, as required in the Declaration of Helsinki.

Participants were positioned in supine position and breath normally for approximately 7 min in a bed-based ballistocardiography setup (see detailed setup description in Carlson et al.^14^). Ballistocardiographs (BCG) were obtained through electromechanical films (EMFit; L series; 300 mm × 580 mm). The analog signals from the film sensors were amplified and bandpass filtered between 0.3 and 24 Hz. LabVIEW (v14.01, National Instruments) digitalized the signals at a sampling rate of 1 kHz. A three-lead ECGs and impedance respiration were acquired using Datex Ohmeda CardioCap (General Electric). Reconstructed brachial artery pressure (reBAP, in kPa), and stroke volume (SV, in dL) were acquired with a Finapres Medical Systems Finometer PRO^®^. Stroke volumes were calculated by proprietary software of the Finometer^®^, using the ModelFlow method after correcting for age, sex, weight, and height. Signal synchronization ensured delays at most by 15 ms. reBAP and SV are model-based reconstructions from finger photoplethysmography measurements, whose implementation and validation can be found elsewhere^52–54^.

Matlab R2022b was used for signal processing. BCGs were bandpass filtered between 1 and 10 Hz to reduce noise and to minimize the contribution of respiration components. Heartbeat strength (HBS) was computed per each heartbeat as the root mean squared of the BCG signal up to 500 ms with respect to the R peak. reBAPs was lowpass filtered with a cutoff frequency of 10 Hz. ECGs were bandpass filtered between 1 and 40 Hz. reBAP and SV signals have an overall delay of 1 s, which was adjusted prior analysis.

### Cardiac rhythmicity descriptors

We used our recently developed and validated approach to quantify cardiac rhythms in a time-resolved manner^31,32,55,56^.

The IBI series was constructed based on the R-to-R-peak durations. Poincaré plot was used to depict the fluctuations on the duration of consecutive IBI^57^. We quantified three features from Poincaré plot: baseline cardiac duration (CCD), cardiac parasympathetic index (CPI) and cardiac sympathetic index (CSI), which correspond to the distance to the origin, and the ratios of the ellipsoid representing the short- and long-term fluctuations of heart rate variability, respectively^58^.

The time-varying fluctuations of the distance to the origin and the ellipse ratios were computed with a sliding-time window, as shown in Eqs. ( 3)–(5):

3 $${{CCD}}\,(t)=\sqrt{{{{mean}}({{{IBI}}}_{i,\ldots ,{{\rm{n}}}-1})}^{2}\,+\,{{{mean}}({{{IBI}}}_{i+1,\ldots ,{{\rm{n}}}})}^{2}\,}$$

4 $${CPI}(t)=\sqrt{{\lambda }_{{\varOmega }_{t}}(1)}$$

5 $${{\mathrm{CSI}}}(t)=\sqrt{{\lambda }_{{\varOmega }_{t}}(2)}$$

where ${\lambda }_{{\varOmega }_{t}}$ is the matrix with the eigenvalues of the covariance matrix of ${{\mathrm{IBI}}}_{{{\rm{i}}},\ldots ,{{\rm{n}}}-1}$ and ${{\mathrm{IBI}}}_{{{\rm{i}}}+1,\ldots ,{{\rm{n}}}}$, with ${\varOmega }_{t}:\,t\,-\,T\,\le \,{t}_{i}\,\le \,t$, and $n$ is the length of IBI in the time window ${\varOmega }_{t}.\,$The robust approach used computes the covariance matrix using a shrinkage covariance estimator based on the Ledoit-Wolf lemma for analytic calculation of the optimal shrinkage intensity^59^.

Note that CSI and CPI, when not corrected by the instantaneous heart rate, are correlated with the low and high frequency (LF and HF) bands of the HRV spectrum^31^. It is important to mention that CSI and CPI do not require a frequency band definition, but rather capture the two main rhythms, that typically fall within LF ( < 0.15 Hz) and HF ( > 0.15 Hz). While this approach has the advantage of identifying two parallel oscillators, it does not allow the search of intermediate frequency bands.

The second-order Poincaré plot fluctuations were quantified as heart rate acceleration-deceleration balance (*Acc*), heart rate arrhythmic behavior (*Arr*), and entropy on acceleration and deceleration transitions (*SPE*)^32^, with a sliding-time window, as shown in Eqs. ( 6)–(8). These measures are based on the number of data points present in each quadrant. The first quadrant of this plot shows the cases of two consecutive heart rate decelerations, while the third quadrant shows two consecutive accelerations. The second and fourth quadrants indicate cases where a deceleration is followed by an acceleration, and vice versa, respectively.

6 $${Acc}(t)={P}_{{\varOmega }_{t}}({Q}_{3})-{P}_{{\varOmega }_{t}}({Q}_{1})$$

7 $${Arr}(t)={P}_{{\varOmega }_{t}}({Q}_{2})-{P}_{{\varOmega }_{t}}({Q}_{4})$$

8 $${{\rm{SPE}}}(t)=\mathop{\sum }\limits_{i=1}^{4}\left(-\frac{{L}_{{\varOmega }_{t}}({Q}_{i})}{{N}_{{\varOmega }_{t}}}{\log }_{2}\frac{{L}_{{\varOmega }_{t}}({Q}_{i})}{{N}_{{\varOmega }_{t}}}\right)$$

Where ${P}_{{\varOmega }_{t}}({Q}_{i})$ is the dispersion of the quadrant, defined as two standard deviations of the Euclidean distance to the origin of all the points part of the quadrant ${Q}_{i}$. ${L}_{{\varOmega }_{t}}({Q}_{i})$ indicates the number of points in the quadrant ${Q}_{i}$, and ${N}_{{\varOmega }_{t}}$ is the total points in the plot. These indices are computed within the time window defined as ${\varOmega }_{t}:\,t\,-\,T\,\le \,{t}_{i}\,\le \,t$, with $T$ fixed at 15 s, based on previous simulations in humans^21,31^.

### Modeling and optimization algorithm

We employed an optimization algorithm to determine the optimal parameters for a model predicting HBS (Eqs. (1) and (2)) from a set of physiological signals, including stroke volume (SV), blood pressure (BP), cardiac cycle duration (CCD), heart rate variability (HRV), and breathing phase (ϕ). The optimization was performed individually for each subject using Matlab’s ‘fminsearch’ function, a derivative-free method based on the Nelder-Mead simplex algorithm. The objective function for each subject was defined as the sum of squared differences between the observed HBS values and the values predicted by the model. This objective function is a measure of the model’s fit, with the goal being to minimize this difference by adjusting the model parameters. The algorithm iteratively adjusted the parameters starting from an initial guess c_1_ = 0.01, c_2_ = 0.01, and c_3_ = 0.0001, seeking to minimize the objective function by minimizing the sum of squared errors. The optimization process was controlled by setting specific tolerance levels for both the function value (at 1e−6) and the parameter estimates (at 1e−6), ensuring a precise convergence to the optimal solution.

This approach allowed for the individualized tuning of model parameters, leading to a tailored fit for each subject’s physiological data, thus enhancing the accuracy of the model’s predictions.

To further compare the model estimation using different cardiac descriptors (HRV), we used Spearman correlation coefficients between the modeled and estimated HBS. Additional analyses using Spearman correlation coefficients were performed to quantify relationships between CPI and breathing activity.

To evaluate model performance and compare alternative predictors of HBS (with and without the HRV component), we computed the coefficient of determination (R²), Akaike Information Criterion (AIC), and Bayesian Information Criterion (BIC). Given the observed HBS time series *y* and model predictions *ŷ*, the residual sum of squares was calculated as:

8 $${{SS}}_{{res}}=\mathop{\sum }\limits_{i=1}^{n}{({y}_{i}-\,{\hat{y}}_{i})}^{2}$$

and the total sum of squares as:

9 $${{SS}}_{{tot}}=\mathop{\sum }\limits_{i=1}^{n}{({y}_{i}-\,mean({y}))}^{2}$$

The coefficient of determination was then computed as:

10 $${R}^{2}=1-\frac{{{SS}}_{{res}}}{{{SS}}_{{tot}}}$$

which reflects the proportion of variance in HBS explained by the model. To assess model complexity and fit simultaneously, we also computed the AIC and BIC as^60,61^:

11 $${AIC}={{\rm{n}}}\cdot {{\rm{ln}}}\left(\frac{{{SS}}_{{res}}}{n}\right)+2k$$

12 $${BIC}={{\rm{n}}}\cdot {{\rm{ln}}}\left(\frac{{{SS}}_{{res}}}{n}\right)+k\cdot {{\rm{ln}}}(n)$$

Where *n* is the length of the HBS signal (in samples) and *k* is the number of parameters set in the model. A positive AIC and BIC difference and a negative *R*^2^ difference (model without HRV component, minus full model) were considered as indicators of enhanced modeling performance when incorporating the HRV component.

### Reporting summary

Further information on research design is available in the Nature Portfolio Reporting Summary linked to this article.

## Supplementary information

Description of Additional Supplementary Files

Supplementary Data

Reporting Summary

## Data Availability

All physiological data used in this study are publicly available^14^ through IEEE DataPort (10.21227/77hc-py84). Data to replicate figures are available in the Supplementary Data file.

## Code availability

Codes implementing the methods of this study are available at https://github.com/diegocandiar/robust_hrv.
